# Advertising expenditures on child-targeted food and beverage products in two policy environments in Canada in 2016 and 2019

**DOI:** 10.1371/journal.pone.0279275

**Published:** 2023-01-11

**Authors:** Monique Potvin Kent, Elise Pauzé, Lauren Remedios, David Wu, Julia Soares Guimaraes, Adena Pinto, Mariangela Bagnato, Meghan Pritchard, Mary L’Abbé, Christine Mulligan, Laura Vergeer, Madyson Weippert

**Affiliations:** 1 School of Epidemiology and Public Health, Faculty of Medicine, University of Ottawa, Ottawa, Canada; 2 School of Interdisciplinary Health Sciences, Faculty of Health Sciences, University of Ottawa, Ottawa, Canada; 3 Schulich School of Medicine and Dentistry, University of Western Ontario, Ontario, Canada; 4 Department of Nutritional Sciences, Faculty of Medicine, University of Toronto, Toronto, Canada; Chung Shan Medical University, TAIWAN

## Abstract

**Background:**

The food industry advertises unhealthy foods intended for children which in turn fosters poor diets. This study characterized advertising expenditures on child-targeted products in Canada and compared these expenditures between Quebec, where commercial advertising to children under 13 is restricted, and the rest of Canada, where food advertising to children is self-regulated.

**Methods:**

Advertising expenditures data for 2016 and 2019 for 57 select food categories and five media channels were licensed from Numerator. Products and brands targeted to children were identified based on their nature and the advertising techniques used to promote them. Advertising expenditures were classified as healthy/unhealthy using Health Canada’s nutrient profile model. Expenditures per child capita aged 2–12 years were calculated and expenditures from 2016 were adjusted for inflation. Advertising expenditures were described by media, food category, year, and geographic region.

**Results:**

Overall, $57.2 million CAD was spent advertising child-targeted products in Canada in 2019. Television accounted for 77% of expenditures followed by digital media (18%), and the food categories with the highest expenditures were candy/chocolate (30%) and restaurants (16%). The totality of expenditures (99.9%-100%) in both Quebec and the rest of Canada in 2016 and 2019 were considered ‘unhealthy’. Across all media channels (excluding digital), advertising expenditures were 9% lower in 2019 versus 2016. Advertising expenditures per capita were 32% lower in Quebec ($9.40/capita) compared to the rest of the country ($13.91/capita).

**Conclusion:**

In Canada, millions are spent promoting child-targeted products considered inappropriate for advertising to children. While per capita advertising expenditures for these products are lower in Quebec compared to the rest of Canada, they remain high, suggesting that Quebec’s commercial advertising restrictions directed to children are likely not sufficiently protecting them from unhealthy food advertising.

## Introduction

Since the 1970’s, childhood obesity has become a significant public health crisis worldwide [1]. While obesity rates in Canadian children have declined over the past two decades, childhood obesity continues to remain prevalent amongst Canadian youth [2, 3]. Several studies indicate that the overall diet quality of Canadian children is poor, with many children failing to meet national dietary recommendations [4–6]. Indeed, food and beverages high in fat, sugar, and/or sodium, including those not recommended by Canada’s Food Guide, accounted for 16–24% of all calories consumed by children aged 2 to 13 years in 2015 [5]. These alarming trends raise significant concerns over children’s long-term cardiovascular and metabolic health, as well as the healthcare costs associated with chronic disease [1, 7, 8].

One factor contributing to children’s poor dietary intake is food and beverage marketing [9–11]. The current children’s food marketing environment is largely comprised of high-calorie, low-nutrient food products designed and promoted by food companies to specifically appeal to children [12, 13].A strong evidence base supports the association between exposure to unhealthy foods and a negative impact on children’s dietary attitudes, preferences, and consumption [9–11, 14]. Research in Canada and other economically developed countries has also shown that food and beverage companies target children across multiple media, including print media (e.g., magazines), [15, 16] television, [17] digital media (e.g., viewed in social media, on desktop computers, on smartphones, etc.), [18–21] and out-of-home media (e.g., billboards, signs, transit shelters) [22] as well as other settings such as schools and retail environments [13, 22–24]. Children have emerged as a critical target market for food and beverage companies because of their heavy influence over household purchases and their future as adult consumers [9]. Purchase requests made by young children are most often for foods high in fat and sugar [25]. The sum of the evidence on food marketing highlights the “cascading effects” of exposure to food marketing on the development of positive brand/food attitudes, increased purchase intent and purchases of advertised food products, and excessive caloric intake that could potentially lead to weight gain [26, 27].

Given the likely impact of food and beverage marketing on children’s behaviors and health, [9–11, 14, 26, 27] restrictions to protect children from these harmful effects are a priority in Canada and globally [28, 29]. In Canada, there are currently two different policy environments with regards to food marketing. Across Canada, with the exception of Quebec, advertising is self-regulated by the food industry through the *Canadian Children’s Food and Beverage Advertising Initiative* (CAI) [30]. As part of this voluntary initiative, one restaurant and 14 food and beverage manufacturers agree to not advertise to children under 12 years old in certain media or only advertise products defined as “healthier” [30, 31]. Despite this attempt at regulatory action, research in Canada has concluded that the CAI is largely ineffective at reducing the use of child-directed marketing techniques and limiting children’s exposure to unhealthy food advertising (Potvin Kent et al., unpublished) [32–34]. In the province of Quebec, where French is the primary language, provincial legislation known as the *Consumer Protection Act* (CPA) has prohibited commercial advertising directed to children under 13 years of age since 1980 across a variety of media channels and child settings [35]. Under this legislation, the advertisement of products intended for children is also banned if they are designed to interest children or if they are placed in media or places where many children are likely to be exposed [35].

Assessments of food advertising on television in Quebec have indicated that the CPA has been responsible for some positive effects on advertising to children [36, 37]. As such, Quebec’s CPA has been proposed as a model for national food marketing restrictions being developed and considered in Canada [28, 38]. Older research comparing food advertising to children in Canada’s two policy environments [19, 36, 37] has suggested that products advertised to children in Quebec are marginally healthier than products advertised in Ontario, while the frequency of television and online food marketing remains similar between the two provinces [19, 36, 37]. Differences in the amount and content of food advertising viewed by children have also been observed within Quebec however findings are somewhat mixed (Potvin Kent et al. unpublished) [36]. Older research based on self-reported television viewing data suggested that Anglophone children in Quebec view slightly more food advertisements on television compared to their Francophone counterparts [36]. However, a recent study based on more objective television viewership data from 2019 found that Francophone children aged 2–11 in Montreal (Quebec) viewed substantially more television food advertisements on the 10 most watched stations compared to Anglophone children in Montreal (Potvin Kent et al. unpublished). On the other hand, Francophone children in Montreal in 2019 viewed substantially less food advertisements featuring child-appealing content and on child-appealing stations, suggesting that Francophone children in Quebec are less explicitly targeted by food advertisements (Potvin Kent et al. unpublished).

The food industry invests billions of dollars in youth-targeted food and beverage marketing across a wide variety of media platforms. Currently much of the available food marketing expenditure data comes from the United States, where the Federal Trade Commission (FTC) reported approximately $1 billion was spent in 2009 on marketing foods to children aged 2–11 [39]. Although child-targeted advertising expenditures decreased from 2006 to 2009, the nutritional quality of promoted foods only modestly improved with most expenditures having been allocated to promoting restaurant foods, carbonated beverages, and snack foods [39]. Such food marketing expenditures are a driving force behind the development of obesogenic food environments and underscore the importance of monitoring advertising expenditures as an essential step towards improving the food marketing landscape for children. To date, no published research in Canada has examined advertising expenditures on child-targeted food and beverage products.

To fill this gap, the purpose of this study was to estimate food advertising expenditures on products intended for children and to compare spending on such products between Quebec and the rest of Canada across various media channels. Specifically, this research answered the following questions: 1) How much was spent on the advertising of child-targeted food products per child capita in Canada, in Quebec, and outside Quebec by media and food category?; 2) How much has spending on advertising of child-targeted products in Canada, in Quebec and outside Quebec changed over time?; 3) How much was spent on advertising healthier versus less healthy products?, and 4) Are there differences in expenditures on the advertising of child-targeted products between English and French language media in Quebec? It was hypothesized that spending on the advertising of child-targeted food products has decreased in traditional media (e.g., television) over time and that most spending is dedicated to unhealthy products. It was also hypothesized that per capita advertising expenditures on child-targeted products is lower in Quebec compared to the rest of Canada and is lower in French media in Quebec compared to English media. This assessment may shed light on the impact of the *Consumer Protection Act* and may inform food marketing restrictions in Canada and internationally.

## Materials and methods

### Advertising expenditure data

Data on food advertising expenditures in Canada in 2016 and 2019 were licensed from Numerator, a company that monitors advertising and estimates advertising expenditures across multiple media. Expenditure data were obtained for 57 select food categories and include spending across 94 television stations, 41 radio stations, 6 out-of-home suppliers (e.g., transit shelters, billboards, etc.), 64 newspapers, 95 magazines, and more than 1000 websites accessed on desktops and mobile devices. For outdoor and radio advertising, Numerator estimates advertising expenditures using billing information provided by out-of-home advertising suppliers and radio stations and broadcasters. To estimate advertising expenditures in print and digital media, Numerator collects its own data on advertising (e.g., information on advertised products and ad size, frequency and/or location) and combines this information with data on media use (e.g., publication circulation, website audience). Advertising spending on all newspaper and magazine sections are monitored except for advertisements smaller than 1/16^th^ of the page. In digital media, expenditures capture display and video advertisements viewed on web browsers while using desktop and mobile devices on the top 1,000 websites with the most traffic in Canada (excluding adult websites, social media or any apps or websites that necessitate a login). For television, Numerator estimates advertising expenditures using both data provided by broadcasters and advertising data the company collects itself to. These expenditures estimates capture advertising on broadcast television while advertising on streaming websites and on-demand services are excluded. The 57 select food categories included both unhealthy food categories heavily advertised to children and salutary food categories (e.g., fruit, vegetables, water). The latter were licenced to allow comparisons in advertising expenditures between healthier versus less healthy food products. The 57 select food categories were collapsed by grouping similar products together to form 12 condensed categories. These included: breads, dessert foods, candy and chocolate, breakfast food, dairy products, condiments, entrees, fruits and vegetables, beverages, snacks, restaurants, and miscellaneous. It should be noted that Numerator’s data captures advertising expenditures in Canada’s ten provinces but not in its three Northern territories (i.e., Yukon, Northwest Territories and Nunavut). The advertising estimates also exclude a 15% mark-up applied by advertising agencies and as such are considered estimates of net advertising expenditures. A more detailed description of the 57 licenced food categories, the condensed categories and Numerator’s methodology for estimating advertising expenditures have been published elsewhere [40].

### Definition of child-targeted products

This study examined advertising expenditures on food and beverage products and brands marketed to children, hereafter referred to as “child-targeted products”. The criteria for identifying child-targeted products are described in Table 1. The development of these criteria was informed by research on the marketing techniques employed on the packaging of child-targeted food products in Canadian grocery stores. [41–43]. Generally, products were deemed child-targeted if the product design and marketing techniques used to promote the product on television and/or product packaging suggested it was intended for or being marketed to children. Examples of such products include breakfast cereals and other child-appealing products promoted with anthropomorphic characters, fun-shaped crackers or chocolate, restaurant meals for children, apple sauce or fruit cups, candy-like fruit snacks and other products promoted as children’s lunch snacks and any type of candy. Two independent coders (EP and LR) reviewed all the products and brands listed in Numerator’s advertising expenditures reports for 2016 (n = 3,672) and 2019 (n = 3,627) to identify child-targeted products. To help with this assessment, television food advertising data from 2019 licenced from Numerator and web searches on company and food retailing websites were consulted. The inter-rater reliability between coders was 99.3% and was calculated using the following formula: (1–49 disagreements/7,299) *100. Differences in coding were settled through consensus. Overall, 101 and 148 products or brands were identified as child-targeted in 2016 and 2019, respectively. As shown in Table 2, the most dominant categories across both 2016 and 2019 were candy and chocolate (27.3% and 30.4% respectively), snacks (17.3% and 17.1% respectively), and breakfast food (16.4% and 16.5% respectively).

**Table 1:** Criteria for identifying “child-targeted” products.

Products considered child-targeted	Products not considered child-targeted unless other child-targeted criteria were met
• Restaurant menu items for children • Branded gaming apps advertised by a food company • Brands or sub-brands of products intended for children (e.g., sometimes denoted using words like “junior” or “mini”) • Brands or products that have “children” or “kids” in their name • Products with unusual shapes, names, flavours or colours (e.g., animal crackers, rainbow-coloured cereal) or that have interactive properties • Products generally marketed as lunch snacks for children (e.g., some compartment lunches and yogurt beverages, individual cups of apple sauce, pudding, gelatin dessert or canned fruit, candy-like fruit snacks). • All candy • Chocolate spreads and fun-shaped chocolate (e.g., shaped like animals or eggs) • Child-appealing products (i.e., chocolate, ready-to-eat breakfast cereal, cookies, cakes and other sweet baked goods, snack foods like chips and popcorn and, ice cream, popsicles, and other similar frozen desserts) that: i. Are marketed using spokes characters or other characters that appeal to children (e.g., anthropomorphic animals or foods) **OR** ii. Featured children in more than 50% of their advertisements airing on television in 2019	• Cereal bars • Aunt Jemima, Bob’s Red Mill) • Snack or other foods that contain candy or other products that are deemed child-targeted (e.g., cookie or candy flavored coffee, snack mix containing candy) • Advertising expenditures for charities or contests unless these were tied to child-targeted products or brands • Any other product **Not considered child-targeted:** Advertising expenditures reported for groups of products or brands that are not exclusively tied to child-targeted products (e.g., “Black Diamond Cheese Family”, “Kellogg’s Food Products Image”)

**Table 2:** Number of products or brands considered child-targeted^†^ by product category and subcategory.

	2016			2019		
Food category	Products	Brands	Total	Products	Brands	Total
	n	n	n (%)	n	n	n (%)
**Candy and chocolate**	27	3	30 (27.3)	45	3	48 (30.4)
**Bread**	1	0	1 (0.9)	0	0	0 (0)
**Breakfast food**	17	1	18 (16.4)	25	1	26 (16.5)
Cold cereal	17	1	18 (16.4)	24	1	25 (15.8)
Waffles, pancakes & French toast	0	0	0 (0)	1	0	1 (0.6)
**Beverages**	0	0	0 (0)	2	0	2 (1.3)
Juices, drinks and nectars	0	0	0 (0)	1	0	1 (0.6)
Water	0	0	0 (0)	1	0	1 (0.6)
**Dairy products**	11	0	11 (10.0)	9	0	9 (5.7)
Cheese	4	0	4 (3.6)	4	0	4 (2.5)
Yogurt	7	0	7 (6.4)	5	0	5 (3.2)
**Dessert foods**	10	1	11 (10.0)	21	1	22 (13.9)
Baked goods (i.e.. cakes, cookies, muffins, pies, tarts, sweet pastries)	7	0	7 (6.4)	10	0	10 (6.3)
Ice cream, frozen yogurt and treats	3	1	4 (3.6)	8	1	9 (5.7)
Pudding and flavoured gelatin	0	0	0 (0)	3	0	3 (1.9)
**Fruit and vegetables**	2	0	2 (1.8)	5	0	5 (3.2)
Canned Fruit	2	0	2 (1.8)	4	0	4 (2.5)
Frozen Vegetables (i.e. potatoes)	0	0	0 (0)	1	0	1 (0.6)
**Sweet spreads**	2	0	2 (1.8)	1	0	1 (0.6)
**Restaurants**	15	0	15 (13.6)	14	0	14 (8.9)
Fast food restaurants	2	0	2 (1.8)	10	0	10 (6.3)
Sit-down restaurants	13	0	13 (11.8)	4	0	4 (2.5)
**Snacks**	16	3	19 (17.3)	26	1	27 (17.1)
Crackers	4	0	4 (3.6)	5	0	5 (3.2)
Portable Snacks	11	1	12 (10.9)	11	0	11 (7.0)
Chips and popcorn	1	2	3 (2.7)	10	1	11 (7.0)
**Food manufacturers**	0	1	1 (0.9)	0	4	4 (2.5)
**Total**	101	9	110 (100)	148	10	158 (100)

^†^Based on products from 57 select food categories licenced from Numerator

#### Nutritional analysis

The nutritional information was collected for each child-targeted product. Nutrient data was primarily derived from the University of Toronto’s Food Label Information Program (FLIP), a large database of food packaging label information for over 17,000 food products purchased from three of the largest Canadian retailers (Loblaws, Sobeys, and Metro) [44]. The nutritional composition for any products not found in this database were collected from the following sources, in order of priority: the company’s Canadian website, the website of a Canadian retailer, the company’s American website, the website of an American retailer, or previously collected nutrition data. A similar process was followed for child-targeted restaurant items. For restaurant products in 2016, nutritional information was collected, in order of priority, from the University of Toronto’s Menu-FLIP 2016 dataset (i.e., containing nutritional information for Canadian restaurant foods) [45]., the restaurant’s Canadian website, the restaurant’s American website, or data previously collected. Restaurant items in 2019 were collected from the restaurant’s Canadian website, the restaurant’s American website, Menu-FLIP for 2016, or previously collected data.

The nutrient profile of all products identified as child-targeted was assessed according to Health Canada’s proposed nutrient profile model. Under this nutrient model, food products were classified as “restricted” or “permitted” for advertising to children (hereafter described as ‘unhealthy’ and ‘healthy’, respectively) based on the product’s saturated fat, total sugars, and sodium content. The nutrient profile model only applies to products with added sugar, saturated fat, or sodium and thresholds for each of these nutrients only apply on a nutrient-by-nutrient basis (e.g. sodium threshold only applies if a product contains added sodium). Those that do not contain added free sugars, sodium or fat are automatically considered “permitted” or healthy. The nutrient content of packaged food products is assessed based on standard serving sizes which vary by product type while restaurant entrees are assessed based 100g portions or based on the portion sizes in which they are sold in the case of all other restaurant foods. In the few cases where advertising expenditures were reported by brand, these were either classified based the classification of most related products available in FLIP datasets or this data was deemed missing. For restaurant meal combos, expenditures were classified as “restricted” or unhealthy if at least one of its individual food items was classified as such. Additional information on the nutrient threshold applied by Health Canada’s nutrient profile model is available elsewhere [40].

#### Ethics statement

This study did not involve any human participants therefore research ethics board approval was not needed to conduct this research.

#### Data analysis

Advertising expenditures on child-targeted food products in 2016 and 2019 were tabulated overall, by food category and by media and were assessed at the national-level, for Quebec and the rest of Canada (i.e., excluding Quebec). To make comparisons between expenditures from 2016 and 2019, advertising spending from 2016 was multiplied by 1.0606 to adjust for inflation. This multiplicative factor was determined using the Bank of Canada’s inflation calculator [46]. Advertising expenditures per child capita aged 2–12 years were calculated by dividing advertising expenditures by child population estimates calculated by Statistics Canada [47] to allow comparisons in advertising expenditures between Quebec and the rest of Canada and between expenditures in French and English media in Quebec. The data used to estimate the English- and French-speaking child population in Quebec and calculate per capita expenditures are described in supplemental Tables 1–3. Absolute and relative differences in expenditures were tabulated to characterize differences in spending by geographic region, media language in Quebec and overtime.

**Table 3:** Advertising expenditures on child-targeted products^†^ in 2019, by media and geographic area.

	Quebec (QC)		Rest of Canada (ROC)		QC vs ROC		Total Canada	
	Total expenditures CAD (%)	Expenditures per child capita^‡^ CAD	Total expenditures CAD (%)	Expenditures per child capita^‡^ CAD	Absolute difference Exp/capita CAD	Relative difference%	Total Expenditures CAD (%)	Expenditures per child capita^‡^ CAD
**Television**	7,257,668 (76.7)	7.20	36,775,593 (77.0)	10.70	-3.50	-32.7	44,033,261 (76.9)	9.91
**Radio**	89,323 (0.9)	0.09	356,630 (0.7)	0.10	-0.02	-14.6	445,953 (0.8)	0.10
**Out-of-Home**	417,985 (4.4)	0.41	2,166,881 (4.5)	0.63	-0.22	-34.2	2,584,866 (4.5)	0.58
**Print media**	24,739 (0.3)	0.02	107,072 (0.2)	0.03	-0.01	-21.2	131,811 (0.2)	0.03
**Digital media**	1,677,161 (17.8)	1.68	8,384,053 (17.5)	2.44	-0.76	-31.3	10,061,214 (17.6)	2.26
**Total**	9,466,876 (100)	9.40	47,790,229 (100)	13.91	-4.51	-32.5	57,257,105 (100)	12.89

CAD: Canadian dollars;

^†^Based on products from 57 select food categories licensed from Numerator;

^‡^Expenditure per child capita aged 2–12 years

## Results

### Advertising expenditure by media in 2019

Nationally, a total of $57.2 million was spent advertising child-targeted products in 2019 across all media (Table 3). Television accounted for the largest share of expenditures (76.9%) followed by digital (17.6%) and out-of-home (4.5%) media. Both print media and radio accounted for less than 1% of expenditures. The distribution of advertising expenditures by media in Quebec and the rest of Canada mirrored that of total expenditures in Canada. Advertising expenditures per child capita were 32% lower in Quebec ($9.40/capita) compared to the rest of Canada ($13.91/capita) across all media. The greatest relative and absolute differences in expenditures in Quebec compared to the rest of the country were observed for out-of-home media (-34.2%; -$0.22/capita), television (-32.7%; -$3.50/capita), and digital media (-31.3%; -$0.76/capita).

### Advertising expenditures by food category in 2019

As shown in Table 4, candy, and chocolate (30.1%), restaurants (16.1%), and breakfast foods (15.3%) accounted for the greatest share of total expenditures on child-targeted products nationally. These product categories were the top 3 sources of expenditures in the rest of Canada while the top sources in Quebec were candy and chocolate (25.6%), snacks (15.7%) and breakfast food (15.0%). Expenditures per child capita were lower in Quebec compared to the rest of Canada for most food categories except food manufacturers (+8,173%; +$0.28/capita) and beverages (+53.8%; +$<0.01/capita). Among subcategories, advertising expenditures per capita for puddings and flavoured gelatin (+199%; +$0.26/capita), yogurt (+2.6%; +$0.01/capita), and juices, drinks, and nectars (+54.4%; +$<0.01/capita) were also higher in Quebec compared to the rest of Canada. Conversely, expenditures per child capita for candy and chocolate (-$1.90/capita, -44.2%), restaurants (-$0.95/capita; -41.4%; particularly fast foods), breakfast food (-$0.72/capita; -33.8%; particularly cold cereal), and dessert foods (-$0.55/capita; -35.7%; particularly baked goods and ice cream) among other food categories and subcategories, were lower in Quebec.

**Table 4:** Advertising expenditures on child-targeted products^†^ across all media in 2019 by geographic region and food category.

	Quebec (QC)		Rest of Canada (ROC)		QC vs ROC		Total Canada	
	Total expenditures CAD (%)	Expenditures per child capita^‡^ CAD	Total expenditures CAD (%)	Expenditures per child capita^‡^ CAD	Absolute difference Exp/child CAD	Relative difference %	Total Expenditures CAD (%)	Expenditures per child capita^‡^ CAD
**Candy and chocolate**	2,419,785 (25.6)	2.40	14,790,283 (30.9)	4.31	-1.90	-44.2	17,210,068 (30.1)	3.87
**Breakfast food**	1,424,528 (15.0)	1.41	7,334,482 (15.3)	2.13	-0.72	-33.8	8,759,010 (15.3)	1.97
Cold cereal	1,424,257 (15.0)	1.41	7,329,957 (15.3)	2.13	-0.72	-33.7	8,754,214 (15.3)	1.97
Waffles	271 (<0.01)	<0.01	4,525 (0.01)	<0.01	-<0.01	-79.6	4,796 (0.01)	<0.01
**Beverages**	2,075 (0.02)	<0.01	4,601 (0.01)	<0.01	+<0.01	+53.8	6,676 (0.01)	<0.01
Juices, drinks and nectars	2,075 (0.02)	<0.01	4,583 (0.01)	<0.01	+<0.01	+54.4	6,658 (0.01)	<0.01
Water	0 (0)	0.00	18 (<0.01)	<0.01	-<0.01	-100	18 (<0.01)	<0.01
**Dairy products**	915,939 (9.7)	0.91	3,624,012 (7.6)	1.05	-0.15	-13.8	4,539,951 (7.9)	1.02
Cheese	557,972 (5.9)	0.55	2,434,277 (5.1)	0.71	-0.15	-21.8	2,992,249 (5.2)	0.67
Yogurt	357,967 (3.8)	0.36	1,189,735 (2.5)	0.35	+0.01	+2.6	1,547,702 (2.7)	0.35
**Dessert foods**	1,002,881 (10.6)	1.00	5,315,988 (11.1)	1.55	-0.55	-35.7	6,318,869 (11.0)	1.42
Baked goods	534,007 (5.6)	0.53	3,549,821 (7.4)	1.03	-0.50	-48.7	4,083,828 (7.1)	0.92
Ice cream, frozen yogurt and treats	80,059 (0.8)	0.08	1,322,764 (2.8)	0.39	-0.31	-79.4	1,402,823 (2.5)	0.32
Pudding and flavoured gelatin	388,815 (4.1)	0.39	443,403 (0.9)	0.13	+0.26	+199	832,218 (1.5)	0.19
**Fruit and vegetables**	19,931 (0.2)	0.02	580,688 (1.2)	0.17	-0.15	-88.3	600,619 (1.0)	0.14
Canned Fruit	16,053 (0.2)	0.02	472,297 (1.0)	0.14	-0.12	-88.4	488,350 (0.9)	0.11
Frozen Vegetables	3,878 (0.04)	<0.01	108,391 (0.2)	0.03	-0.03	-87.8	112,269 (0.2)	0.03
**Sweet spreads**	550,846 (5.8)	0.55	2,289,386 (4.8)	0.67	-0.12	-18.0	2,840,232 (5.0)	0.64
**Restaurants**	1,351,489 (14.3)	1.34	7,870,765 (16.5)	2.29	-0.95	-41.4	9,222,254 (16.1)	2.08
Fast food restaurants	1,350,117 (14.3)	1.34	7,805,060 (16.3)	2.27	-0.93	-41.0	9,155,177 (16.0)	2.06
Sit-down restaurants	1,372 (0.01)	<0.01	65,705 (0.1)	0.02	-0.02	-92.9	67,077 (0.1)	0.02
**Snacks**	1,490,756 (15.7)	1.48	5,968,127 (12.5)	1.74	-0.26	-14.8	7,458,883 (13.0)	1.68
Crackers	255,583 (2.7)	0.25	1,012,815 (2.1)	0.29	-0.04	-13.9	1,268,398 (2.2)	0.29
Portable Snacks	134,982 (1.4)	0.13	710,715 (1.5)	0.21	-0.07	-35.2	845,697 (1.5)	0.19
Chips and popcorn	1,100,191 (11.6)	1.09	4,244,597 (8.9)	1.23	-0.14	-11.6	5,344,788 (9.3)	1.20
**Food manufacturers**	288,646 (3.0)	0.29	11,897 (0.02)	<0.01	+0.28	+8,173	300,543 (0.5)	0.07

CAD: Canadian dollars;

^†^Based on products from 57 select food categories licensed from Numerator;

^‡^Expenditure per child capita aged 2–12 years

### Healthfulness of advertising expenditures in 2016 and 2019

The near totality of advertising expenditures on child-targeted products nationally in both 2016 and 2019 was for unhealthy products (S4 and S5 Tables). In 2016, all spending in Quebec and the rest of Canada was devoted to unhealthy products. In 2019, the share of advertising expenditures classified as unhealthy were marginally higher in Quebec (99.97%) compared to the rest of Canada (99.95%).

### Changes in expenditures between 2016 and 2019

In Canada overall, per capita advertising expenditures on child-targeted products were 11.6% lower in 2019 compared to 2016 across all media (excluding digital), going from $12.02/capita to $10.62/capita. National advertising expenditures per child capita in 2019 compared to 2016 were lower for television (-13.0%) and print media (-92.8%) and higher for out-of-home (+191.6%) and radio (+331.5%) (Table 5). The same temporal differences in per capita advertising expenditures were noted in Quebec and the rest of Canada however the magnitude of these differences varied by media between these two geographic regions. Most notably, between 2016 and 2019, per capita expenditures on radio and out-of-home media increased by 30.4% and 46.3% respectively in Quebec. In comparison, across the rest of Canada, per capita expenditures in these media increased by 891.6% and 259.4% respectively between 2016 and 2019. The relative decreases in per capita expenditures on television and print media were relatively similar in Quebec (-10.3% and -94.9% respectively) and the rest of Canada (-13.3% and -92.1% respectively).

**Table 5:** Advertising expenditures on child-targeted products^†^ in 2016 and 2019, by geographic area and media (excluding digital).

	Total expenditures CAD (%)		Absolute difference CAD	% change	Expenditures per child capita^‡^ CAD		Absolute difference CAD	% change
	2016^§^	2019			2016^§^	2019		
	**Quebec**							
**Television**	7,694,855 (90.6)	7,257,668 (93.2)	-437,187	-5.7	8.03	7.20	-0.83	-10.3
**Radio**	65,174 (0.8)	89,323 (1.1)	+24,149	+37.1	0.07	0.09	+0.02	+30.4
**Out-of-Home**	271,730 (3.2)	417,985 (5.4)	+146,255	+53.8	0.28	0.41	+0.13	+46.3
**Print media**	464,033 (5.5)	24,739 (0.3)	-439,294	-94.7	0.48	0.02	-0.46	-94.9
**Total**	8,495,791 (100)	7,789,715 (100)	-706,076	-8.3	8.87	7.73	-1.13	-12.8
	**Rest of Canada**							
**Television**	41,384,142 (95.5)	36,775,593 (93.3)	-4,608,549	-11.1	12.35	10.70	-1.64	-13.3
**Radio**	35,090 (0.1)	356,630 (0.9)	+321,540	+916.3	0.01	0.10	+0.09	+891.6
**Out-of-Home**	588,280 (1.4)	2,166,881 (5.5)	1,578,601	+268.3	0.18	0.63	+0.46	+259.4
**Print media**	1,318,070 (3.0)	107,072 (0.3)	-1,210,998	-91.9	0.39	0.03	-0.36	-92.1
**Total**	43,325,582 (100)	39,406,176 (100)	-3,919,406	-9.0	12.93	11.47	-1.46	-11.3
	**Total Canada**							
**Television**	49,078,997 (94.7)	44,033,261 (93.3)	-5,045,736	-10.3	11.39	9.91	-1.48	-13.0
**Radio**	100,264 (0.2)	445,953 (0.9)	+345,689	+344.8	0.02	0.10	+0.08	+331.5
**Out-of-Home**	860,010 (1.7)	2,584,866 (5.5)	+1,724,856	+200.6	0.20	0.58	+0.38	+191.6
**Print media**	1,782,103 (3.4)	131,811 (0.3)	-1,650,292	-92.6	0.41	0.03	-0.38	-92.8
**Total**	51,821,373 (100)	47,195,891 (100)	-4,625,482	-8.9	12.02	10.62	-1.40	-11.6

CAD: Canadian dollars;

^†^Based on products from 57 select food categories licensed from Numerator;

^‡^Expenditure per child capita aged 2–12 years;

^§^Expenditures adjusted for inflation

Temporal changes in per capita advertising expenditures by food category were observed (S6–S8 Tables). Nationally, per capita expenditures between 2016 and 2019 increased the most in relative terms for food manufacturers (+117,004%; +$0.06/capita) and in absolute terms for dessert foods (+$0.58/capita; +99.9%) while decreasing the most in relative terms for bread (-100%; -$0.09) and in absolute terms for snacks (-$0.76/capita; -38.2%). Similar temporal differences in per capita expenditures by food category were also observed in Quebec and the rest of Canada, with some exceptions. In Quebec, per capita expenditures for sweet spreads decreased by 47.6% while increasing by 16.2% for the rest of Canada. Per capita advertising expenditures for restaurants and food manufacturers were also 12.6% and 3,914,047% higher, respectively, in 2019 compared to 2016 in Quebec yet these expenditures were 1.1% and 100% lower, respectively, in 2019 compared to 2016 in the rest of Canada. Additionally, temporal changes in expenditures varied in magnitude between Quebec and the rest of Canada for most food categories. For instance, decreases in per capita expenditures were smaller in Quebec for snacks (-22.3%) and breakfast food (-4.6%) and higher for chocolate (-28.2%) and fruit and vegetables (-94.1%) compared to the rest of Canada (-40.7%, -26.5%, -9.1% and -45.4% respectively). Increases in per capita expenditures were also greater in Quebec for dessert food (+177%) and fast-food restaurants (+37.7%) and lower for yogurt (+21.2%) compared to the rest of Canada (+94%, +5.0% and +60.1%).

### Advertising expenditures in French and English media in 2019

Overall, $1.82 million and $7.66 million or $12.80 and $8.62 per child capita was spent advertising child-targeted food products in English and French language media, respectively, in Quebec in 2019 (Table 6). The distribution of advertising expenditures by media were comparable between French and English media. Of total expenditures in 2019, 73% and 77.5% was allocated to advertising child-targeted products on television in English media and French media, respectively. This was followed by digital media, which accounted for 25% of expenditures in English media and 16.1% in French media. Overall advertising expenditures per child capita were 48.5% higher in English media compared to French media. Per capita advertising expenditures were also higher in all English media channels including television (+$2.67/capita; +40.0%), digital media (+$1.82/capita; +131%), radio (+$0.12/capita; +173%) and print media (+0.05$/capita; +253%), except for out-of-home media, where there were no advertising expenditures for child-targeted food products (-$0.47/capita; -100%).

**Table 6:** Advertising expenditures on child-targeted products^†^ in Quebec in 2019, by language.

	English Media (ENG)		French Media (FR)		ENG vs FR Media	
	Total expenditures CAD (%)	Expenditures per Anglophone child capita^‡^ CAD	Total expenditures CAD (%)	Expenditures per Francophone child capita^‡^ CAD	Absolute difference Exp/child CAD	Relative difference%
**Television**	1,327,224 (73.0)	9.34	5,930,445 (77.5)	6.67	+ 2.67	+40.0
**Radio**	27,115 (1.5)	0.19	62,208 (0.8)	0.07	+0.12	+172.7
**Out-of-Home**	0 (0)	0.00	417,985 (5.5)	0.47	-0.47	-100
**Print media**	8,931 (0.5)	0.06	15,808 (0.2)	0.02	+0.05	+253
**Digital media**	454,660 (25.0)	3.20	1,230,075 (16.1)	1.38	+1.82	+131
**Total**	1,817,930 (100)	12.80	7,656,521 (100)	8.62	+4.18	+48.5

CAD: Canadian dollars;

^†^Based on products from 57 select food categories licensed from Numerator;

^‡^Expenditure per child capita aged 2–12 years

In English media, advertising expenditures on child-targeted food products were the highest for candy and chocolate (32.4%), breakfast food (14.7%), and snacks (14.7%) and the lowest expenditures were on juices, drinks, and nectars (<0.1%), food manufacturers (<0.1%), fruits and vegetables (1.1%) and sit-down restaurants (<0.1%) (Table 7). Similarly, candy and chocolate (23.9%), snacks (16%), and breakfast food (15.1%) were the greatest source of advertising expenditures in French media, and the lowest expenditures were on juices, drinks, and nectars (<0.1%), fruits and vegetables (<0.1%), and food manufacturers (3.8%). Advertising expenditures per child capita were higher in English media for most food categories and subcategories compared to French media, except for juices, drinks and, nectars (-<$0.01/capita; -26.2%), yogurt (-$0.05/capita; -14.6%), pudding and flavoured gelatin (-$0.07/capita; -185%), and food manufacturers (-$0.32/capita; -98.8%). Among per capita advertising expenditures that were higher in English media compared to French media, the greatest relative differences were for fruit and vegetables (+83,514%, particular canned fruit), waffles (+2,009%), and candy and chocolate (+101%) while the greatest absolute differences were for candy and chocolate (+$2.09/capita), breakfast food (+$0.58/capita; particularly cold cereal), and snacks (+$0.50/capita; particularly chips and popcorn).

**Table 7:** Advertising expenditures on child-targeted products^†^ in Quebec in 2019, by language and food category.

	English Media (ENG)		French Media (FR)		ENG vs FR Media	
	Total expenditures CAD (%)	Expenditures per Anglophone child capita^‡^ CAD	Total expenditures CAD (%)	Expenditures per Francophone child capita^‡^ CAD	Absolute difference Exp/child CAD	Relative difference %
**Candy and chocolate**	589,337 (32.4)	4.15	1,830,226 (23.9)	2.06	+2.09	+101
**Breakfast food**	267,708 (14.7)	1.88	1,156,785 (15.1)	1.30	+0.58	+44.8
Cold cereal	267,499 (14.7)	1.88	1,156,723 (15.1)	1.30	+0.58	+44.7
Waffles	209 (<0.1)	<0.01	62 (<0.1)	<0.01	+<0.01	+2,009
**Juices, drinks, and nectars**	219 (<0.1)	<0.01	1,856 (<0.1)	<0.01	-<0.01	-26.2
**Dairy products**	132,151 (7.3)	0.93	783,788 (10.2)	0.88	+0.05	+5.5
Cheese	89,171 (4.9)	0.63	468,801 (6.1)	0.53	+0.10	+19.0
Yogurt	42,980 (2.4)	0.30	314,987 (4.1)	0.35	-0.05	-14.6
**Dessert foods**	197,567 (10.9)	1.39	805,311 (10.5)	0.91	+0.48	+53.5
Baked goods	122,443 (6.7)	0.86	411,568 (5.4)	0.46	+0.40	+86.1
Ice cream, frozen yogurt and treats	30,330 (1.7)	0.21	49,729 (0.6)	0.06	+0.16	+281
Pudding and flavoured gelatin	44,794 (2.5)	0.32	344,014 (4.5)	0.39	-0.07	-185
**Fruit and vegetables**	19,783 (1.1)	0.14	148 (<0.1)	<0.01	+0.14	+83,514
Canned Fruit	15,905 (0.9)	0.11	148 (<0.1)	<0.01	+0.11	+67,124
Frozen vegetables (i.e. potatoes)	3,878 (0.2)	0.03	0 (0)	0.00	+0.03	-
**Sweet spreads**	99,087 (5.5)	0.70	451,759 (5.9)	0.51	+0.19	+37.2
**Restaurants**	244,647 (13.5)	1.72	1,114,677 (14.6)	1.25	+0.47	+37.3
Fast food restaurants	244,328 (13.4)	1.72	1,113,624 (14.5)	1.25	+0.47	+37.2
Sit-down restaurants	319 (<0.1)	<0.01	1,053 (<0.1)	0.00	+<0.01	+89.5
**Snacks**	266,888 (14.7)	1.88	1,223,868 (16.0)	1.38	+0.50	+36.4
Crackers	37,237 (2.0)	0.26	218,346 (2.9)	0.25	+0,02	+6.7
Portable Snacks	18,745 (1.0)	0.13	116,237 (1.5)	0.13	+0,00	+0.9
Chips and popcorn	210,906 (11.6)	1.48	889,285 (11.6)	1.00	+0,48	+47.9
**Food manufacturers**	543 (<0.1)	<0.01	288,103 (3.8)	0.32	-0.32	-98.8

CAD: Canadian dollars;

^†^Based on products from 57 select food categories licensed from Numerator;

^‡^Expenditure per child capita aged 2–12 years

## Discussion

### Summary of main findings

This study found that advertising expenditures for products targeted to children across Canada are high and most of these expenditures were allocated to the promotion of products considered less healthy. Overall, these expenditures decreased between 2016 and 2019, including on broadcast television and other traditional media, as expected. Consistent with our study hypotheses, per child capita advertising expenditures on child-targeted products were lower in Quebec compared to the rest of Canada and lower in French media in Quebec than in English media.

### Nature and healthfulness of child-targeted products

Our study found that advertising expenditures on child-targeted products were higher for less healthy food categories, such as chocolate and candy, fast food restaurants, and breakfast food (particularly cold cereal) while advertising spending for healthier food categories, such as fruit and vegetables and water, were markedly lower. Moreover, almost all advertising expenditures on examined products in Quebec and the rest of Canada were considered ‘unhealthy’ or restricted for advertising to children by the nutrient profile model proposed by Health Canada. These findings are consistent with research from the United States, which suggests that advertisers continue to spend heavily on the advertisement of food and beverages of low nutritional quality towards youth [48]. This is of particular concern as unhealthy food advertising is recognized as a likely potent contributor to poor diet and a myriad of related downstream health consequences [9–11, 26, 49]. The high percentage of advertising expenditures for restricted or unhealthy products that was observed in this study further underscores the prominence of spending devoted to less healthy foods and beverages. However, these results are based on classifications using a nutrient profile model proposed by Health Canada, which categorizes products based on stringent nutrient content thresholds for total sugars, saturated fat, and sodium. A previous comparison of nutrient profile models suggests that the Health Canada model was the most stringent, and that under this model only 3% of 374 child-targeted products in Canadian supermarkets were permitted to be advertised to children [50]. In addition, our criteria for identifying child-targeted products somewhat favoured unhealthy products as some criteria (i.e. use of child-appealing characters and children in television advertising) only applied to certain unhealthy food categories that are thought to be particularly appealing to children. Despite these potential sources of bias, the predominance of spending devoted to unhealthy food products relative to healthier ones is consistent with research on child-directed food advertising spending in the United States and reflective of the low frequency of healthy food advertising on child-appealing television stations and websites in Canada (Potvin Kent et al. unpublished) [32].

### Differences in per child capita expenditures between regions

As hypothesized, this study observed lower per capita expenditures on child-targeted products in Quebec compared to the rest of Canada across all media platforms and for most food categories, except for juices, yogurt, pudding, and food manufacturers. Within Quebec, total advertising expenditures on child-targeted products were also lower in French media compared to English media. It is unclear whether these noted differences in advertising expenditures are reflective of differences in food advertising frequency as the cost of advertising may differ between geographic regions or between English and French language media in Quebec. Nevertheless, these findings are consistent with available research on food advertising on television which has found differences in the nature or content of food advertisements viewed by Anglophone children in Ontario and Francophone and Anglophone children in Quebec (Potvin Kent et al. unpublished) [36]. For instance, Francophone children in Quebec view fewer advertisements featuring child-appealing advertising techniques and fewer ads on child-appealing stations suggesting that they may be less targeted by products explicitly advertised to and intended for children (Potvin Kent et al. unpublished) [36].

Although advertising per capita is lower in Quebec compared to the rest of Canada and in French compared to English media, overall advertising expenditures are still high in Quebec as a whole, and particularly in English media. The gaps in the *Consumer Protection Act* are most apparent for English children in Quebec, for whom exposure to unhealthy food advertising continues to be comparable to English children in Ontario (where self-regulation exists) (Potvin Kent et al. unpublished). When considered together, these findings suggest that Quebec’s *Consumer Protect Act* does not entirely limit advertising for products intended for or targeted to children. For instance, advertising expenditures were still allocated to the promotion of candy, children’s fast food meals, and portable lunch snacks in Quebec. Most recently in February 2022, an Act, Bill C-252, to amend the Food and Drugs Act (prohibiting food and beverage advertising directed at children) was introduced in the Canadian House of Commons [51]. This bill proposes restricting all advertising of products high in sugar, saturated fats, or sodium directed to children under 13 years old. Should this bill pass, it would likely help reduce advertising expenditures for child-targeted products across Canada and within Quebec, given that nearly all products examined in this study were considered subject to marketing restrictions.

### Differences in per capita expenditures over time

The results of this study indicate an overall decline in per capita expenditures across media platforms in Quebec and the rest of Canada between 2016 and 2019. While most advertising expenditures on child-targeted products were allocated to television in both 2016 and 2019 and in both geographic regions, per capita advertising decreased in traditional media including on television, radio and print media between examined time periods. These temporal changes may reflect changes in advertising costs overtime. Moreover, such changes in expenditures in traditional media may point to shifts in media preferences among children. Digital media consumption is growing among children, with almost 25% of children spending between 1–2 hours on digital devices for personal use during the week, a number that tends to increase with age [52]. Other recent Canadian statistics have also indicated the increasingly important role that digital media plays in children and youth’s everyday lives [53]. As a result, it is possible that advertising spending is shifting from traditional to digital media. Unfortunately, this study was unable to examine temporal changes in advertising expenditures in digital media as data for digital media was unavailable for 2016.

### Strengths and limitations

This is one of the few studies to estimate food advertising expenditures directed at children and the only study to do so in Canada. Although this study cannot speak to children’s exposure to food advertising, this examination of expenditures provides some insight as to the advertising practices of the food industry, including on the radio and in print and out-of-home media, which have not been examined comprehensively or at all in Canada for food advertising directed to children [54, 55]. Nevertheless, this study’s methodological limitations should be highlighted. First, our study mostly captures advertising expenditures on products intended for children or using advertising techniques that suggest explicit targeting of children and does not include expenditures on products that appeal to children and wider audiences (e.g., chocolate, soft drinks) that are not advertised in a manner that targets children explicitly. As such, this study likely underestimated how much is spent on food advertising to children in Canada. Second, our comparison of advertising spending using per capita expenditures assumes that all children within a geographic region or media market consumes a given media. Given that food companies likely allocate their advertising dollars based on the audience known to be reached by a given media, calculating per capita expenditures based on child audience measures may have yielded better estimates of per capita expenditures. Unfortunately, media audience measures are not publicly available. Third, our identification of child-targeted products relied in part on the content of advertisements airing on broadcast television in 2019. Given that we did not have such data for 2016, some child-targeted products may have been missed therefore advertising expenditures may have been underestimated in 2016. Finally, there are some limitations that are inherent in the data licensed from Numerator. Estimates of advertising expenditures for television are limited to broadcast television and do not include advertising spending allocated to on-demand television services and streaming on broadcaster websites or other platforms. Advertising expenditures in digital media were also only available for 2019 and only included video advertising appearing at the beginning of videos and display advertising viewed on the 1,000 most visited websites with advertising in Canada, excluding those that necessitate a login like social media among others. Consequently, digital media had to be excluded from temporal comparisons and Numerator’s data likely underestimates advertising expenditures in digital media. It should also be noted that trend analyses were not conducted to examine changes in advertising over time, Numerator’s expenditure estimates do not include advertising in Canada’s northern territories, and our findings only apply to the 57 select food categories included in the study.

## Conclusion

This study revealed that advertising spending on unhealthy child-targeted products across Canada is high and that differences in per capita advertising expenditures are apparent between Quebec and the rest of Canada. Further research corroborating expenditure data with children’s exposure to such products is needed to evaluate Quebec’s food advertising restrictions. Continued monitoring of advertising expenditures on child-targeted products is also recommended.

## Supporting information

S1 Table Estimated number of children aged 2–12 years old by geographic region and year. ^†^The estimated number of children aged 2–12 excludes those living in Yukon, Nunavut, and the Northwest Territories. Data Source: Statistics Canada.
(DOCX)

S2 Table Percentage of children aged 2–12 years old in Quebec whose first official language is French, English, French and English or neither French and English. Data Source: Statistics Canada, based on 2016 census, custom tabulation.
(DOCX)

S3 Table Estimated number of children aged 2–12 years old in Quebec whose first language is English and French in 2019. Note: Estimated by multiplying the estimated number of children in Quebec in 2019 by the percentage of children who speak English and French as a first official language.
(DOCX)

S4 Table Advertising expenditures on child-targeted products^†^ across all media (excluding digital media) in 2016 by Health Canada’s proposed nutrient profile model (NPM) classification and by geographic region. (DOCX)

S5 Table Advertising expenditures on child-targeted products^†^ across all media (including digital media) in 2019 by Health Canada’s proposed nutrient profile model (NPM) classification and by geographic region. (DOCX)

S6 Table Differences in advertising expenditures on child-targeted products† in Canada between 2016 and 2019 by food category. CAD: Canadian dollars; ^†^Based on products from 57 select food categories licensed from Numerator; ^‡^Expenditure per child capita aged 2–12 years and includes advertising expenditures data for broadcast television, radio, out-of-home, and print media; ^§^Inflation-adjusted expenditures.
(DOCX)

S7 Table Differences in advertising expenditures on child-targeted products^†^ in Quebec between 2016 and 2019 by food category. CAD: Canadian dollars; ^†^Based on products from 57 select food categories licensed from Numerator and includes advertising expenditures data for broadcast television, radio, out-of-home, and print media; ^‡^Expenditure per child capita aged 2–12 years; ^§^Inflation-adjusted expenditures.
(DOCX)

S8 Table Differences in advertising expenditures on child-targeted products^†^ in the rest of Canada between 2016 and 2019 by food category. CAD: Canadian dollars; ^†^Based on products from 57 select food categories licensed from Numerator and includes advertising expenditures data for broadcast television, radio, out-of-home, and print media; ^‡^Expenditure per child capita aged 2–12 years; ^§^Inflation-adjusted expenditures.
(DOCX)

## List of abbreviations

NPM: Nutrient Profile Model
FLIP: Food Label Information Program
CAD: Canadian dollars
QC: Quebec
ROC: Rest of Canada
