# Supplementary material for: Advertising expenditures on child-targeted food and beverage products in two policy environments in Canada in 2016 and 2019
Source: PLoS One. 2023 Jan 11;18(1):e0279275. doi: 10.1371/journal.pone.0279275 (PMC9833551; doi:10.1371/journal.pone.0279275)
Supplement: S1 Table — †The estimated number of children aged 2–12 excludes those living in Yukon, Nunavut, and the Northwest Territories. Data Source: Statistics Canada. (DOCX) [file pone.0279275.s001.docx]

**S1 Table. Estimated number of children aged 2-12 years old by geographic region and year.**

|  | **Quebec** | **Rest of Canada**^†^ | **Total Canada**^†^ |
| --- | --- | --- | --- |
| **2016** | 958,273 | 3,351,958 | 4,310,231 |
| **2019** | 1,007,478 | 3,435,513 | 4,442,991 |

^†^The estimated number of children aged 2-12 excludes those living in Yukon, Nunavut, and the Northwest Territories

**Data Source:** Statistics Canada
