# Supplementary material for: Advertising expenditures on child-targeted food and beverage products in two policy environments in Canada in 2016 and 2019
Source: PLoS One. 2023 Jan 11;18(1):e0279275. doi: 10.1371/journal.pone.0279275 (PMC9833551; doi:10.1371/journal.pone.0279275)
Supplement: S2 Table — Data Source: Statistics Canada, based on 2016 census, custom tabulation. (DOCX) [file pone.0279275.s002.docx]

**S2 Table. Percentage of children aged 2-12 years old in Quebec whose first official language is French, English, French and English or neither French and English.**

|  | **%** |
| --- | --- |
| **English** | 10.7 |
| **French** | 84.8 |
| **French and English** | 3.4 |
| **Other language** | 1.1 |

**Data Source:** Statistics Canada, based on 2016 census, custom tabulation
