# Supplementary material for: Advertising expenditures on child-targeted food and beverage products in two policy environments in Canada in 2016 and 2019
Source: PLoS One. 2023 Jan 11;18(1):e0279275. doi: 10.1371/journal.pone.0279275 (PMC9833551; doi:10.1371/journal.pone.0279275)
Supplement: S3 Table — Note: Estimated by multiplying the estimated number of children in Quebec in 2019 by the percentage of children who speak English and French as a first official language. (DOCX) [file pone.0279275.s003.docx]

**S3 Table. Estimated number of children aged 2-12 years old in Quebec whose first language is English and French in 2019.**

|  | **First language**  **% of children aged 2-11** | **n** |
| --- | --- | --- |
| **French** | 88.2 | 888,596 |
| **English** | 14.1 | 142,054 |

**Note:** Estimated by multiplying the estimated number of children in Quebec in 2019 by the percentage of children who speak English and French as a first official language
