# Supplementary material for: Advertising expenditures on child-targeted food and beverage products in two policy environments in Canada in 2016 and 2019
Source: PLoS One. 2023 Jan 11;18(1):e0279275. doi: 10.1371/journal.pone.0279275 (PMC9833551; doi:10.1371/journal.pone.0279275)
Supplement: S4 Table — (DOCX) [file pone.0279275.s004.docx]

**S4 Table. Advertising expenditures on child-targeted products^†^ across all media (excluding digital media) in 2016 by Health Canada’s proposed nutrient profile model (NPM) classification and by geographic region**

|  | **Quebec** | **Rest of Canada** | **Total Canada** |
| --- | --- | --- | --- |
|  | **Expenditures**  **CAD (%)** | **Expenditures**  **CAD (%)** | **Expenditures**  **CAD (%)** |
| **Healthier/Permitted** | 0 (0) | 0 (0) | 0 (0) |
| **Less healthy/Restricted** | 8,440,790 (100) | 42,928,218 (100) | 51,369,008 (100) |
| **Total spending classified by NPM** | 8,440,790 (100) | 42,928,218 (100) | 51,369,008 (100) |
|  |  |  |  |
| **Spending not classified by the NPM (% of total expenditures)** | 55,001 (0.6) | 397,364 (0.9) | 452,365 (0.9) |
