# Supplementary material for: Advertising expenditures on child-targeted food and beverage products in two policy environments in Canada in 2016 and 2019
Source: PLoS One. 2023 Jan 11;18(1):e0279275. doi: 10.1371/journal.pone.0279275 (PMC9833551; doi:10.1371/journal.pone.0279275)
Supplement: S5 Table — (DOCX) [file pone.0279275.s005.docx]

**S5 Table. Advertising expenditures on child-targeted products^†^ across all media (including digital media) in 2019 by Health Canada’s proposed nutrient profile model (NPM) classification and by geographic region**

|  | **Quebec** | **Rest of Canada** | **Total Canada** |
| --- | --- | --- | --- |
|  | **Expenditures**  **CAD (%)** | **Expenditures**  **CAD (%)** | **Expenditures**  **CAD (%)** |
| **Healthier/Permitted** | 2,802 (0.03) | 23,020 (0.05) | 25,822 (0.05) |
| **Less healthy/Restricted** | 9,463,816 (99.97) | 47,737,451 (99.95) | 57,201,267 (99.95) |
| **Total spending classified by NPM** | 9,466,618 (100) | 47,760,471 (100) | 57,227,089 (100) |
|  |  |  |  |
| **Spending not classified by the NPM (i.e. missing data)** | 258 (<0.01) | 29,758 (<0.06) | 30,016 (0.05) |
