# Supplementary material for: Advertising expenditures on child-targeted food and beverage products in two policy environments in Canada in 2016 and 2019
Source: PLoS One. 2023 Jan 11;18(1):e0279275. doi: 10.1371/journal.pone.0279275 (PMC9833551; doi:10.1371/journal.pone.0279275)
Supplement: S6 Table — CAD: Canadian dollars; †Based on products from 57 select food categories licensed from Numerator; ‡Expenditure per child capita aged 2–12 years and includes advertising expenditures data for broadcast television, radio, out-of-home, and print media; §Inflation-adjusted expenditures. (DOCX) [file pone.0279275.s006.docx]

**S6 Table.** **Differences in advertising expenditures on child-targeted products^†^ in Canada between 2016 and 2019 by food category.**

|  | **Total expenditures**  **CAD (%)** | | **Absolute difference** | **% change** | **Expenditures per child capita**^‡^  **CAD** | | **Absolute difference** | **% change** |
| --- | --- | --- | --- | --- | --- | --- | --- | --- |
|  | **2016**^§^ | **2019** |  |  | **2016**^§^ | **2019** |  |  |
| **Candy and chocolate** | 13,710,809 (26.5) | 12,340,883 (26.1) | -1,369,926 | -10,0 | 3.18 | 2.78 | -0.40 | -12.7 |
| **Bread** | 406,938 (0.8) | 0 (0) | -406,938 | -100 | 0.09 | 0.00 | -0.09 | -100 |
| **Breakfast food** | 10,344,245 (20.0) | 8,136,009 (17.2) | -2,208,236 | -21,3 | 2.40 | 1.83 | -0.57 | -23.7 |
| Cold cereal | 10,344,245 (20.0) | 8,136,009 (17.2) | -2,208,236 | -21,3 | 2.40 | 1.83 | -0.57 | -23.7 |
| Waffles | 0 (0) | 0 (0) | 0 | - | 0 | 0 | **-** | **-** |
| **Beverages** | 0 (0) | 0 (0) | 0 | - | 0 | 0 | **-** | **-** |
| Juices, drinks and nectars | 0 (0) | 0 (0) | 0 | - | 0 | 0 | **-** | **-** |
| Water | 0 (0) | 0 (0) | 0 | - | 0 | 0 | **-** | **-** |
| **Dairy products** | 4,083,780 (7.9) | 4,137,943 (8.8) | +54,163 | +1.3 | 0.95 | 0.93 | -0.02 | -1.7 |
| Cheese | 3,155,542 (6.1) | 2,715,165 (5.8) | -440,377 | -14.0 | 0.73 | 0.61 | -0.12 | -16.5 |
| Yogurt | 928,238 (1.8) | 1,422,778 (3.0) | +494,540 | +53.3 | 0.22 | 0.32 | +0.10 | +48.7 |
| **Dessert foods** | 2,516,797 (4.9) | 5,185,436 (11.0) | +2,668,639 | +106.0 | 0.58 | 1.17 | +0.58 | +99.9 |
| Baked goods | 2,437,391 (4.7) | 3,856,735 (8.2) | +1,419,344 | +58.2 | 0.57 | 0.87 | +0.30 | +53.5 |
| Ice cream, frozen yogurt and treats | 79,406 (0.2) | 1,328,701 (2.8) | +1,249,295 | +1573 | 0.02 | 0.30 | +0.28 | +1,523 |
| Pudding and flavoured gelatin | 0 (0) | 0 (0) | 0 | - | 0 | 0 | **-** | **-** |
| **Fruit and vegetables** | 1,351,203 (2.6) | 598,413 (1.3) | -752,790 | -55.7 | 0.31 | 0.13 | -0.18 | -57.0 |
| Canned Fruit | 1,351,203 (2.6) | 486,144 (1.0) | -865,059 | -64.0 | 0.31 | 0.11 | -0.20 | -65.1 |
| Frozen Vegetables (i.e. potatoes) | 0 (0) | 112,269 (0.2) | +112,269 | - | 0,00 | 0.03 | +0.03 | - |
| **Sweet spreads** | 2,754,452 (5.3) | 2,657,001 (5.6) | -97,451 | -3.5 | 0.64 | 0.60 | -0.04 | -6.4 |
| **Restaurants** | 8,114,997 (15.7) | 8,413,689 (17.8) | +298,692 | +3.7 | 1.88 | 1.89 | +0.01 | +0.6 |
| Fast food restaurants | 7,457,035 (14.4) | 8,355,313 (17.7) | +898,278 | +12.0 | 1.73 | 1.88 | +0.15 | +8.7 |
| Sit-down restaurants | 657,962 (1.3) | 58,376 (0.1) | -599,586 | -91.1 | 0.15 | 0.01 | -0.14 | -91.4 |
| **Snacks** | 8,537,912 (16.5) | 5,438,458 (11.5) | -3,099,454 | -36.3 | 1.98 | 1.22 | -0.76 | -38.2 |
| Crackers | 1,973,761 (3.8) | 1,175,701 (2.5) | -798,060 | -40.4 | 0.46 | 0.26 | -0.19 | -42.2 |
| Portable Snacks | 2,211,258 (4.3) | 705,379 (1.5) | -1,505,879 | -68.1 | 0.51 | 0.16 | -0.35 | -69.1 |
| Snack food | 4,352,893 (8.4) | 3,557,378 (7.5) | -795,515 | -18.3 | 1.01 | 0.80 | -0.21 | -20.7 |
| **Food manufacturers** | 239 (0) | 288,059 (0.6) | +287,820 | +120,611 | <0.01 | 0.06 | +0.06 | +117,004 |

CAD: Canadian dollars; ^†^Based on products from 57 select food categories licensed from Numerator and includes advertising expenditures data for broadcast television, radio, out-of-home, and print media; ^‡^Expenditure per child capita aged 2-12 years; ^§^Inflation-adjusted expenditures
