# Supplementary material for: Advertising expenditures on child-targeted food and beverage products in two policy environments in Canada in 2016 and 2019
Source: PLoS One. 2023 Jan 11;18(1):e0279275. doi: 10.1371/journal.pone.0279275 (PMC9833551; doi:10.1371/journal.pone.0279275)
Supplement: S7 Table — CAD: Canadian dollars; †Based on products from 57 select food categories licensed from Numerator and includes advertising expenditures data for broadcast television, radio, out-of-home, and print media; ‡Expenditure per child capita aged 2–12 years; §Inflation-adjusted expenditures. (DOCX) [file pone.0279275.s007.docx]

**S7 Table. Differences in advertising expenditures on child-targeted products^†^ across in Quebec between 2016 and 2019 by food category.**

|  | **Total expenditures**  **CAD (%)** | | **Absolute difference** | **% change** | **Expenditures per child capita**^‡^  **CAD** | | **Absolute difference** | **% change** |
| --- | --- | --- | --- | --- | --- | --- | --- | --- |
|  | **2016**^§^ | **2019** |  |  | **2016**^§^ | **2019** |  |  |
| **Candy and chocolate** | 2,446,264 (28.8) | 1,846,785 (23.7) | -599,479 | -24.5 | 2.55 | 1.83 | -0.72 | -28.2 |
| **Bread** | 20,255 (0.2) | 0 (0) | -20,255 | -100 | 0.02 | 0.00 | -0.02 | -100 |
| **Breakfast food** | 1,379,764 (16.2) | 1,383,612 (17.8) | +3,848 | +0.3 | 1.44 | 1.37 | -0.07 | -4.6 |
| Cold cereal | 1,379,764 (16.2) | 1,383,612 (17.8) | +3,848 | +0.3 | 1.44 | 1.37 | -0.07 | -4.6 |
| Waffles | 0 (0) | 0 (0) | 0 | - | 0 | 0 | - | - |
| **Beverages** | 0 (0) | 0 (0) | 0 | - | 0 | 0 | - | - |
| Juices, drinks and nectars | 0 (0) | 0 (0) | 0 | - | 0 | 0 | - | - |
| Water | 0 (0) | 0 (0) | 0 | - | 0 | 0 | - | - |
| **Dairy products** | 874,008 (10.3) | 876,973 (11.3) | +2,965 | +0.3 | 0.91 | 0.87 | -0.04 | -4.6 |
| Cheese | 600,337 (7.1) | 528,178 (6.8) | -72,159 | -12.0 | 0.63 | 0.52 | -0.10 | -16.3 |
| Yogurt | 273,671 (3.2) | 348,795 (4.5) | +75,124 | +27.5 | 0.29 | 0.35 | +0.06 | +21.2 |
| **Dessert foods** | 195,228 (2.3) | 569,432 (7.3) | +374,204 | +192 | 0.20 | 0.57 | +0.36 | +177 |
| Baked goods | 191,994 (2.3) | 492,776 (6.3) | +300,782 | +157 | 0.20 | 0.49 | +0.29 | +144 |
| Ice cream, frozen yogurt and treats | 3,234 (0) | 76,656 (1.0) | +73,422 | +2,271 | <0.01 | 0.08 | +0.07 | +2,155 |
| Pudding and flavoured gelatin | 0 (0) | 0 (0) | 0 | - | 0 | 0 | - | - |
| **Fruit and vegetables** | 317,920 (3.7) | 19,694 (0.3) | -298,226 | -93.8 | 0.33 | 0.02 | -0.31 | -94.1 |
| Canned Fruit | 317,920 (3.7) | 15,816 (0.2) | -302,104 | -95.0 | 0.33 | 0.02 | -0.32 | -95.3 |
| Frozen Vegetables (i.e. potatoes) | 0 (0) | 3,878 (0.05) | +3,878 | - | 0 | <0.01 | +<0.01 | - |
| **Sweet spreads** | 973,830 (11.5) | 536,303 (6.9) | -437,527 | -44.9 | 1.02 | 0.53 | -0.48 | -47.6 |
| **Restaurants** | 1,088,022 (12.8) | 1,287,568 (16.5) | +199,546 | +18.3 | 1.14 | 1.28 | +0.14 | +12.6 |
| Fast food restaurants | 889,465 (10.5) | 1,287,568 (16.5) | +398,103 | +44.8 | 0.93 | 1.28 | +0.35 | +37.7 |
| Sit-down restaurants | 198,557 (2.3) | 0 (0) | -198,557 | -100 | 0.21 | 0.00 | -0.21 | -100 |
| **Snacks** | 1,200,492 (14.1) | 981,289 (12.6) | -219,203 | -18.3 | 1.25 | 0.97 | -0.28 | -22.3 |
| Crackers | 266,206 (3.1) | 243,447 (3.1) | -22,759 | -8.5 | 0.28 | 0.24 | -0.04 | -13.0 |
| Portable Snacks | 159,464 (1.9) | 128,154 (1.6) | -31,310 | -19.6 | 0.17 | 0.13 | -0.04 | -23.6 |
| Snack food | 774,821 (9.1) | 609,688 (7.8) | -165,133 | -21.3 | 0.81 | 0.61 | -0.20 | -25.2 |
| **Food manufacturers** | 7 (<0.01) | 288,059 (3.7) | +288,052 | +4,115,029 | <0.01 | 0.29 | +0.29 | +3,914,047 |

CAD: Canadian dollars; ^†^Based on products from 57 select food categories licensed from Numerator and includes advertising expenditures data for broadcast television, radio, out-of-home, and print media; ^‡^Expenditure per child capita aged 2-12 years; ^§^Inflation-adjusted expenditures
