# Supplementary material for: Advertising expenditures on child-targeted food and beverage products in two policy environments in Canada in 2016 and 2019
Source: PLoS One. 2023 Jan 11;18(1):e0279275. doi: 10.1371/journal.pone.0279275 (PMC9833551; doi:10.1371/journal.pone.0279275)
Supplement: S8 Table — CAD: Canadian dollars; †Based on products from 57 select food categories licensed from Numerator and includes advertising expenditures data for broadcast television, radio, out-of-home, and print media; ‡Expenditure per child capita aged 2–12 years; §Inflation-adjusted expenditures. (DOCX) [file pone.0279275.s008.docx]

**S8 Table.** **Differences in advertising expenditures on child-targeted products^†^ in the rest of Canada between 2016 and 2019 by food category.**

|  | **Total expenditures**  **CAD (%)** | | **Absolute difference** | **% change** | **Expenditures per child capita**^‡^  **CAD** | | **Absolute difference** | **% change** |
| --- | --- | --- | --- | --- | --- | --- | --- | --- |
|  | **2016**^§^ | **2019** |  |  | **2016**^§^ | **2019** |  |  |
| **Candy and chocolate** | 11,264,545 (26.0) | 10,494,098 (26.6) | -770,447 | -6.8 | 3.36 | 3.05 | -0.31 | -9.1 |
| **Bread** | 386,683 (0.9) | 0 (0) | -386,683 | -100 | 0.12 | 0.00 | -0.12 | -100 |
| **Breakfast food** | 8,964,481 (20.7) | 6,752,397 (17.1) | -2,212,084 | -24.7 | 2.67 | 1.97 | -0.71 | -26.5 |
| Cold cereal | 8,964,481 (20.7) | 6,752,397 (17.1) | -2,212,084 | -24.7 | 2.67 | 1.97 | -0.71 | -26.5 |
| Waffles | 0 (0) | 0 (0) | - | - | 0 | 0 | - | - |
| **Beverages** | 0 (0) | 0 (0) | - | - | 0 | 0 | - | - |
| Juices, drinks and nectars | 0 (0) | 0 (0) | - | - | 0 | 0 | - | - |
| Water | 0 (0) | 0 (0) | - | - | 0 | 0 | - | - |
| **Dairy products** | 3,209,772 (7.4) | 3,260,970 (8.3) | +51,198 | +1.6 | 0.96 | 0.95 | -0.01 | -0.9 |
| Cheese | 2,555,205 (5.9) | 2,186,987 (5.5) | -368,218 | -14.4 | 0.76 | 0.64 | -0.13 | -16.5 |
| Yogurt | 654,567 (1.5) | 1,073,983 (2.7) | +419,416 | +64.1 | 0.20 | 0.31 | +0.12 | +60.1 |
| **Dessert foods** | 2,321,570 (5.4) | 4,616,004 (11.7) | +2,294,434 | +98.8 | 0.69 | 1.34 | +0.65 | +94.0 |
| Baked goods | 2,245,397 (5.2) | 3,363,959 (8.5) | +1,118,562 | +49.8 | 0.67 | 0.98 | +0.31 | +46.2 |
| Ice cream, frozen yogurt and treats | 76,172 (0.2) | 1,252,045 (3.2) | +1,175,873 | +1,544 | 0.02 | 0.36 | +0.34 | +1,504 |
| Pudding and flavoured gelatin | 0 (0) | 0 (0) | - | - | 0 | 0 | - | - |
| **Fruit and vegetables** | 1,033,283 (2.4) | 578,719 (1.5) | -454,564 | -44.0 | 0.31 | 0.17 | -0.14 | -45.4 |
| Canned Fruit | 1,033,283 (2.4) | 470,328 (1.2) | -562,955 | -54.5 | 0.31 | 0.14 | -0.17 | -55.6 |
| Frozen Vegetables (i.e. potatoes) | 0 (0) | 108,391 (0.3) | +108,391 | - | 0 | 0.03 | +0.03 | - |
| **Sweet spreads** | 1,780,622 (4.1) | 2,120,698 (5.4) | +340,076 | +19.1 | 0.53 | 0.62 | +0.09 | +16.2 |
| **Restaurants** | 7,026,976 (16.2) | 7,126,121 (18.1) | +99,145 | +1.4 | 2.10 | 2.07 | -0.02 | -1.1 |
| Fast food restaurants | 6,567,570 (15.2) | 7,067,745 (17.9) | +500,175 | +7.6 | 1.96 | 2.06 | +0.10 | +5.0 |
| Sit-down restaurants | 459,405 (1.1) | 58,376 (0.1) | -401,029 | -87.3 | 0.14 | 0.02 | -0.12 | -87.6 |
| **Snacks** | 7,337,420 (16.9) | 4,457,169 (11.3) | -288,0251 | -39.3 | 2.19 | 1.30 | -0.89 | -40.7 |
| Crackers | 1,707,554 (3.9) | 932,254 (2.4) | -775,300 | -45.4 | 0.51 | 0.27 | -0.24 | -46.7 |
| Portable Snacks | 2,051,793 (4.7) | 577,225 (1.5) | -1,474,568 | -71.9 | 0.61 | 0.17 | -0.44 | -72.6 |
| Snack food | 3,578,072 (8.3) | 2,947,690 (7.5) | -630,382 | -17.6 | 1.07 | 0.86 | -0.21 | -19.6 |
| **Food manufacturers** | 231 (<0.01) | 0 (0) | -231 | -100 | <0.01 | 0.00 | -<0.01 | -100 |

CAD: Canadian dollars; ^†^Based on products from 57 select food categories licensed from Numerator and includes advertising expenditures data for broadcast television, radio, out-of-home, and print media; ^‡^Expenditure per child capita aged 2-12 years; ^§^Inflation-adjusted expenditures
